# CL316,243, a β3-adrenergic receptor agonist, induces muscle hypertrophy and increased strength

**DOI:** 10.1038/srep37504

**Published:** 2016-11-22

**Authors:** Daniela Puzzo, Roberto Raiteri, Clotilde Castaldo, Raffaele Capasso, Ester Pagano, Mariateresa Tedesco, Walter Gulisano, Lisaveta Drozd, Pellegrino Lippiello, Agostino Palmeri, Pietro Scotto, Maria Concetta Miniaci

**Affiliations:** 1 Department of Biomedical and Biotechnological Sciences - Section of Physiology, University of Catania, Catania, Italy; 2 Department of Informatics, Bioengineering, Robotics, and System Engineering, University of Genova, Italy; 3 Department of Public Health, School of Medicine and Surgery, University of Naples Federico II, Naples, Italy; 4 Department of Pharmacy, University of Naples Federico II, Naples, Italy

## Abstract

Studies *in vitro* have demonstrated that β3-adrenergic receptors (β3-ARs) regulate protein metabolism in skeletal muscle by promoting protein synthesis and inhibiting protein degradation. In this study, we evaluated whether activation of β3-ARs by the selective agonist CL316,243 modifies the functional and structural properties of skeletal muscles of healthy mice. Daily injections of CL316,243 for 15 days resulted in a significant improvement in muscle force production, assessed by grip strength and weight tests, and an increased myofiber cross-sectional area, indicative of muscle hypertrophy. In addition, atomic force microscopy revealed a significant effect of CL316,243 on the transversal stiffness of isolated muscle fibers. Interestingly, the expression level of mammalian target of rapamycin (mTOR) downstream targets and neuronal nitric oxide synthase (NOS) was also found to be enhanced in tibialis anterior and soleus muscles of CL316,243 treated mice, in accordance with previous data linking β3-ARs to mTOR and NOS signaling pathways. In conclusion, our data suggest that CL316,243 systemic administration might be a novel therapeutic strategy worthy of further investigations in conditions of muscle wasting and weakness associated with aging and muscular diseases.

Studies in humans and animal models have revealed that β-adrenergic receptors (β-ARs) stimulation exerts potent anabolic effects on striated muscles^1^,^2^. Since activation of β-ARs induces skeletal muscle growth associated, in some cases, with an increase of contractile function^3^,^4^, β-AR agonists have been proposed as a therapeutic intervention to counteract muscle wasting correlated with aging or chronic diseases such as muscle dystrophy^5^,^6^,^7^. However, the potential for targeting β-ARs in dystrophies has been diminished because of the mild improvements in skeletal mass/function and adverse cardiac events induced by β1/β2 ARs agonists^2^. So far, much of our knowledge on the role of β-AR signaling in skeletal muscle is based on studies focused on β2-AR agonists, since β2-AR is considered the predominant subtype in skeletal muscle^2^. However, β3-ARs have been also identified in human and rodent skeletal muscles^8^,^9^. Selective activation of β3-ARs has been established to determine important metabolic responses in skeletal muscle such as glucose uptake, phosphorylation, and oxidation leading to an increase of energy expenditure^10^. In addition, β3-AR agonists have been shown to affect muscle thermogenesis by increasing the expression of the uncoupling protein-3 (UCP-3), a protein that uncouples mitochondrial respiration from ATP production, thereby dissipating energy in the form of heat^11^. Even though metabolic effects of β3-AR activation are highly recognized, less is known about the impact of β3-ARs in the regulation of skeletal muscle structure and function. Using a β3-AR selective agonist, CL316,243, we have recently demonstrated *in vitro* that β3-ARs play a critical role in the regulation of protein metabolism in skeletal muscle^12^. In particular, we found that CL316,243 induced a significant increase of skeletal muscle constitutive proteins into muscle cell proteins such as myosin heavy chain, myosin light chain, and actin in rat L6 myocytes. Such anabolic effect was associated with the activation of PI3K/Akt/mTOR pathway, via Gi/o protein, resulting in an increase of p70S6 kinase (p70^S6K^) and protein translation. Another signaling pathway that has been linked to β3-AR is the G protein inhibitory (Gi)–nitric oxide (NO) pathway^13^. In ventricular muscles, activation of the β3-AR receptors by BRL 37344 is accompanied by decreased contractility via NO production. The β3-AR-induced negative inotropic effect was shown to be inhibited by the NOS inhibitor L-NAME and could be reversed by an excess of the NOS-substrate, L-arginine^14^.

Based on these lines of evidence, we first examined whether the *in vivo* administration of the β3-AR agonist CL316,243 affected skeletal muscle strength in adult mice. By using atomic force microscopy (AFM), we next determined whether β3-AR stimulation modifies the mechanical properties of dissociated skeletal muscle fibers. Furthermore, to gain more insight into the molecular mechanism underlying the β3-AR function in skeletal muscle, we investigated whether CL316,243 treatment was associated with an upregulation of the putative β3-AR signaling transduction pathways, involving p70^S6K^ as well as the neuronal nitric oxide synthase (nNOS), which is considered the main source of NO in skeletal muscle^15^.

## Results

### CL316,243 treatment induces an increase in skeletal muscle strength in adult healthy mice

**Figure 1:**
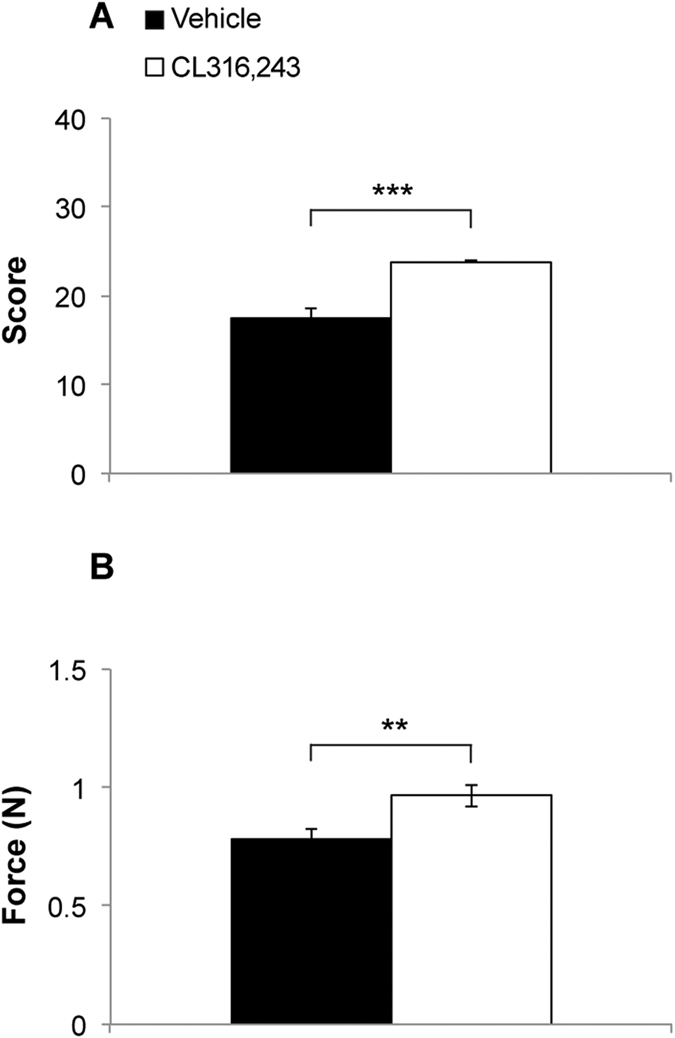
Effects of treatment with CL316,243 on muscular strength in wild type mice. (**A**) Mice treated with the β3-AR agonist CL316,243 show an increase in the strength score. The score was calculated as the product of the number of links in the heaviest chain held for the full 3 sec, multiplied by the time (sec) it was held (n = 10 CL316,243-treated mice vs n = 9 vehicle-treated mice, unpaired t-test: t_(17)_ = 5.495, p < 0.0001). (**B**) Grip test shows an increase in peak force in CL316,243-treated mice compared to vehicle (n = 10/9; unpaired t-test: t_(17)_ = 2.978, p = 0.008). ***p < 0.0001, **p < 0.01.

Muscular strength was assessed in wild-type healthy mice treated with the selective β3-ARs agonist CL316,243 (CL; 1 mg/kg) or saline once per day for 15 days. As shown in Fig. 1A, CL-treated mice exhibited a significant increase in the strength score on the weight test (23.9 ± 0.1 vs. 17.44 ± 1.23; p < 0.0001). These results were confirmed by the grip strength test, showing that CL316,243 treatment resulted in a 23% increase of peak force with respect to control (0.96 ± 0.04 vs. 0.78 ± 0.03; p = 0.008; Fig. 1B). Furthermore, we found that injections of CL316,243 at this dose and duration did not affect mice body weight (corresponding to 27.81 ± 0.31 grams before CL-treatment vs. 27.93 ± 0.30 grams after CL-treatment; t_(17)_ = 0.275, p = 0.787).

### CL316,243 regulates the mechanical properties of skeletal muscle fibers

**Figure 2:**
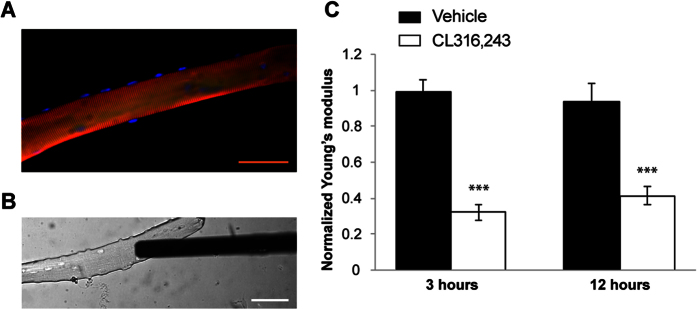
Effects of CL316,243 treatment on muscle stiffness. (**A**) Immunofluorescence image of a dissociated FDB muscle fiber to visualize Z-bands (red), F-actin (green) and nuclei (blue). (**B**) Bright field image of a single FDB fiber and the AFM rectangular cantilever during nanoindentation measurements. **(C)** Normalized stiffness indicated as Young’s modulus, was obtained by AFM nanoindentation measurements from single dissociated muscle fibers, at 200 nm penetration depth. Fibers were incubated with CL316,243 or vehicle and stiffness was measured after 3 or 12 h treatment (n = 4–5 fibers/treatment, two-way ANOVA for treatment F_(1,14)_ = 81.022, p < 0.0001, time F_(1,14)_ = 0.087; p = 0.772, and treatment x time interaction F_(1,14)_ = 1.218; p = 0.288). ***p < 0.0001. Calibration bar = 100 μm.

To determine whether β3-AR activation can also affect the mechanical properties of the cytoskeleton, we measured the transversal stiffness of CL316,243-treated flexor digitorum brevis (FDB) fibers in the relaxed state by means of AFM-based nanoindentation technique. AFM is a useful tool for studying cell mechanics since it allows to apply controlled loads in the nanoNewtown range to living cells and measure the corresponding cell deformation with nanometer resolution^16^,^17^,^18^ (Fig. 2A and B). AFM measurements were performed on single dissociated muscle fibers incubated with either control solution or 1 μM CL316,243 for 3 or 12 hours. As shown in Fig. 2C, treatment with CL316,243 induced a significant reduction of transversal stiffness in the sub-sarcolemma region, at an indentation depth of 200 nm, when compared to untreated fibers at both time points (p < 0.0001).

### CL316,243 treatment induces an increase in muscle fiber cross-sectional area in adult healthy mice

**Figure 3:**
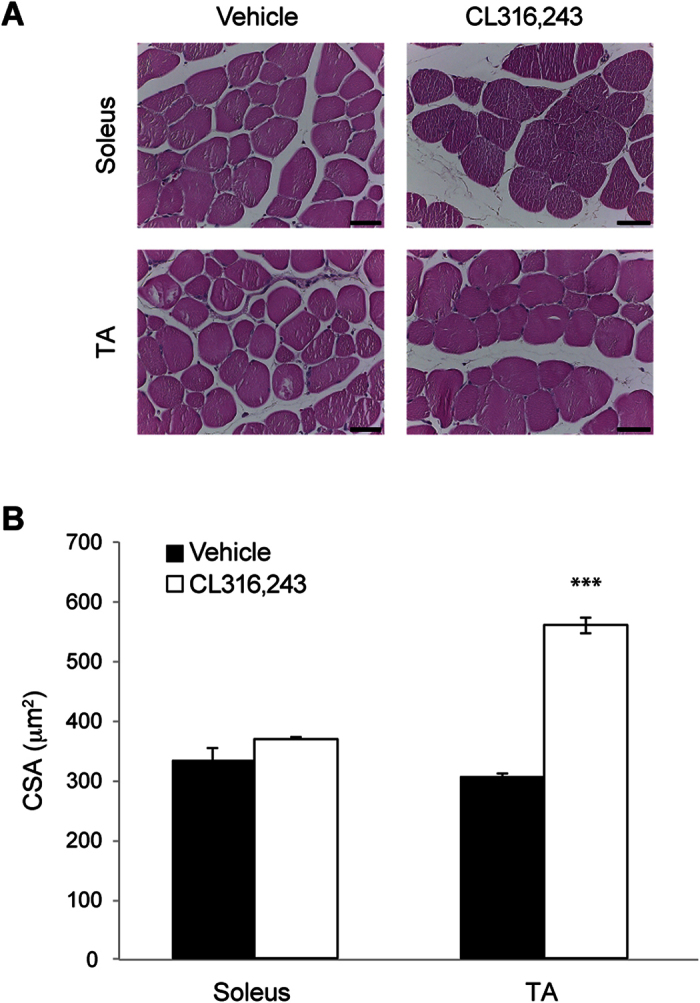
Effects of CL316,243 treatment on fiber cross-sectional area. (**A**) Representative images of hematoxylin and eosin staining of soleus (upper panels) and TA (bottom panels) muscles from mice treated with CL316,243 or vehicle. The stained muscle sections were analyzed for CSA. (**B**) Bar Graph showing the increase in TA CSA after treatment with CL316,243 compared to vehicle (TA CSA, n = 3–4 samples/treatment; unpaired t-test: t_(5)_ = 15.08, p < 0.0001; soleus CSA n = 3 soleus muscle samples/treatment; unpaired t-test: t_(4)_ = 1.86; p = 0.13). ***p < 0.0001. Calibration bar = 20 μm.

To assess whether the increase in muscle strength induced by CL316,243 was associated with muscle hypertrophy, we measured the cross-sectional area (CSA) of hindlimb of CL-treated mice. For this purpose, we selected the soleus and the tibialis anterior (TA), as representative of slow-twitch and fast-twitch muscles, respectively. Morphometric analysis of muscle fibers revealed that CL316,243 induced a significant increase by 83% in TA muscle fiber CSA (p < 0.0001), whereas in soleus CSA was slightly increased by 11% (p = 0.13) compared to control (Fig. 3A and B). The examined muscles did not show any sign of degeneration such as intracytoplasmic vacuoles and centralized nuclei.

### CL316,243 treatment increases the skeletal muscle expression level of p70^S6K^ and rpS6

**Figure 4:**
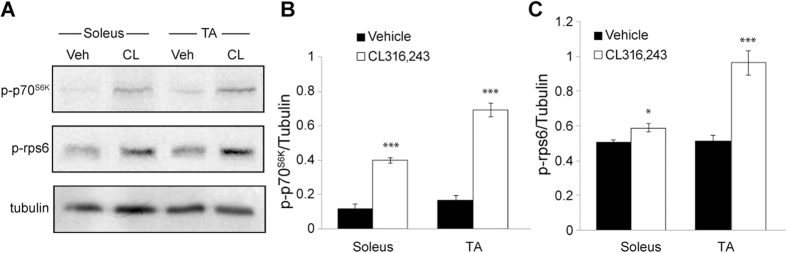
Effects of CL316,243 treatment on phospho-p70^S6K^ and phospho-rpS6 expression levels in skeletal muscles. (**A**) Representative western blot showing the p-p70^S6K^ and p-rpS6 protein expression in TA and soleus skeletal muscles of mice treated with CL316,243 (CL) or vehicle (Veh). α-Tubulin was used as internal loading control. **(B**) Densitometric quantification of protein shows significant increase of of p-p70^S6K^ in both soleus and TA after treatment with CL316,243 (n = 5/5; for soleus unpaired t-test: t_(8)_ = 8.51, p < 0.0001; for TA unpaired t-test: t_(8)_ = 6.04, p < 0.0001). (**C**) Densitometric analysis shows significant increase of p-rpS6 p70^S6K^ in both soleus and TA after treatment with CL316,243 (n = 5/5; for soleus unpaired t-test: t_(8)_ = 2.93, p = 0.019; for TA unpaired t-test: t_(8)_ = 5.91, p < 0.0001). *p < 0.05, ***p < 0.0001.

According to our previous studies *in vitro*, β3-ARs stimulation up-regulates protein synthesis in myocyte cultures and this effect is likely mediated by the PI3K– mTOR- p70^S6K^ signaling pathway activation. Indeed, the CL316,243-induced increase of p70^S6K^ was markedly inhibited by wortmannin, a PI3K inhibitor, and rapamycin, a specific inhibitor of mTOR^12^. Based on these observations, we examined whether the *in vivo* administration of CL316,243 modulates the expression of p70^S6K^ and its downstream target, rpS6, in skeletal muscles obtained from mice treated with CL316,243 or vehicle. As shown in Fig. 4A and B, western blot analysis revealed that the expression level of phospho-p70^S6K^ was significantly higher in both TA and soleus muscles of CL316,243-treated mice with respect to vehicle-treated mice (p < 0.0001). This up-regulation of p70^S6K^ was associated with an increased expression of phospho-rpS6 in TA (p < 0.0001) and soleus (p < 0.05) when compared to control conditions (Fig. 4A and C).

### CL316,243 treatment increases the skeletal muscle expression level of neuronal-NOS

**Figure 5:**
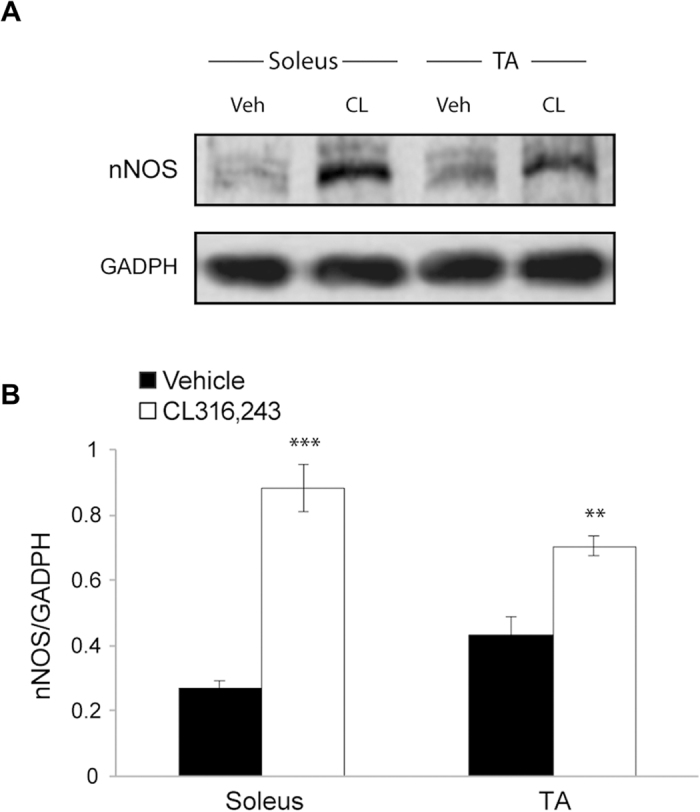
Effects of CL316,243 treatment on nNOS expression levels in skeletal muscles. (**A**) Representative western blot showing the nNOS protein expression in TA and soleus skeletal muscles of mice treated with CL316,243 (CL) or vehicle (Veh). GADPH was used as internal loading control. (**B**) Densitometric quantification of protein shows significant increase of nNOS in both soleus and TA after treatment with CL316,243 (n = 5/5; for soleus unpaired t-test: t_(8)_ = 8.41, p < 0.0001; for TA unpaired t-test: t_(8)_ = 4.57, p = 0.002). **p < 0.01, ***p < 0.0001.

Studies in murine myocardium have demonstrated that application of the β3-AR agonist, BRL 37344, modulates NOS activity and increases NO formation^19^. Additional evidence for nNOS coupling to β3-AR comes from studies showing that the β3-AR-induced negative inotropic effect is absent in cardiomyocytes of nNOS-deficient (NOS1−/−) mice as well as in control cardiomyocytes with acute nNOS inhibition^20^. We, therefore, examined whether CL316,243 treatment *in vivo* affected the expression level of nNOS, which is the most abundant NOS isoform in skeletal muscle. As shown in Fig. 5A and B, nNOS appeared to be regulated in both TA (p < 0.01) and soleus (p < 0.0001) of CL316,243-treated mice with respect to vehicle-treated mice. These data suggest that nNOS may be involved in the β3-AR effects on skeletal muscle.

## Discussion

This study provides the first demonstration that β3-AR activation has important anabolic effects on skeletal muscle. In particular, we show that CL316,243 treatment induces a significant increase of muscle CSA and strength in adult healthy mice compared to controls. The increase in CSA, indicative of muscle hypertrophy, was particularly evident in TA compared to soleus muscle, suggesting a difference in response to β3-agonists between fast- and slow-twitch skeletal muscles. The increase of CSA in TA, a muscle containing a high quantity of fast-contracting fibers, is in agreement with several studies reporting a hypertrophic effect of β2-AR agonist mainly in fast-twitch fibers^21^,^22^. Conversely, there is conflicting evidence concerning whether the slow-contracting fibers are affected by β2-AR agonist treatment in intact animals^23^. In addition, when mice were challenged with specific behavioral tests to evaluate skeletal muscle function, we found an increase of muscle strength in CL316,243-treated mice compared to vehicle-treated animals. Such positive effect was confirmed by the grip strength test, which allowed us to evaluate the peak resistance force, and by the weights test, evaluating the maximal isometric strength.

Several agents have been shown to increase skeletal muscle mass and force^24^ by regulating protein synthesis, including anabolic steroids, growth hormones, IGF and β2-AR agonists^4^,^25^,^26^,^27^,^28^. Most of these factors control the rate of protein turnover at the level of transcription, translation, degradation or a combination of these^29^. According to our previous studies *in vitro*, β3-ARs stimulation up-regulates protein synthesis and this effect is likely due to the activation of components of the translational machinery, including the ribosomal protein S6^12^. Here, the use of an *in vivo* model confirmed our previous *in vitro* data, thus providing a further demonstration that CL316,243 has the potential to regulate protein metabolism in skeletal muscle by increasing the expression of mTOR targets in the long term. The importance of mTOR in muscle size regulation has been demonstrated by both pharmacological and genetic studies^30^,^31^,^32^. For example, inhibition of mTOR by rapamycin prevented the hypertrophy of myotubes induced *in vitro* by IGF as well as the skeletal muscle hypertrophy *in vivo* induced by overload or clenbuterol^25^,^33^,^34^,^35^. A decrease in muscle mass and fiber CSA has also been revealed in mTOR and p70^S6K^ knockout mice^36^,^37^. According to Navegantes and collaborators^38^, the anabolic effects of CL316,243 in skeletal muscles are in part due to an inhibition of muscle proteolysis. Such anti-proteolytic effect was particularly evident in rat soleus, but not in extensor digitorum longus, suggesting a different response to β3-ARs agonist of slow-twitch and fast-twitch muscle. However, the effects of β3-AR activation in slow-twitch muscle fibers are quite complex since they can also involve the regulation of mitochondrial uncoupling proteins. In particular, immunohistochemical studies have revealed an increase of UCP-3 signals in slow-twitch muscles of obese mice following a chronic administration of CL316,243, which may contribute to the thermogenic effect of β3-AR agonists^11^,^39^. In this study, we have also shown that CL316,243 treatment was associated with an increased expression of the nNOS protein in both fast- and slow-twitch muscles. In skeletal muscle, NO has been identified as a physiological intracellular messenger modulating the contractile activity of skeletal muscle, blood flow, exercise-induced skeletal muscle hypertrophy and glucose homeostasis^40^,^41^,^42^,^43^,^44^. The precise role of the nNOS isoform in skeletal muscle is still a matter of debate, although several lines of evidence suggested that NO plays a key role in the hypertrophic response of skeletal muscle to mechanical and metabolic stimulations^45^,^46^. NOS activity has been shown to promote transcription of contractile proteins, such as skeletal α-actin and type I myosin heavy chain mRNA, during chronic skeletal muscle overload^44^. According to our previous studies *in vitro*^47^, the anabolic effect of NOS might be mediated by the mTOR/p70^S6K^ signaling pathway since NOS inhibition by L-NAME prevented the activation of p70^S6K^ in response to glucose deprivation. The importance of nNOS in skeletal muscle has been clearly revealed by studies carried out in nNOS-deficient (NOS1−/−) mice^48^. These mice presented a significant reduction of muscle mass and CSA of tibialis anterior accompanied by a decrease of muscle force and decreased resistance to fatigue, compared with control mice. Interestingly, the TA of NOS1−/− mice showed also lower levels of phosphorylated rpS6, 4E-BP1, and Akt than controls, suggesting that the AKT/mTOR pathway activation was reduced in absence of nNOS. In addition, recent studies have demonstrated that restoration of NO signaling by nNOS overexpression can reduce muscle pathology in mouse models of muscular dystrophy (named mdx mice) by preventing muscle membrane injury and promoting regeneration^49^. A novel important finding of this study is that the acute treatment with CL316,243 induced a significant decrease of the transversal stiffness of single muscle fibers within the first 1000 nm of the fiber surface. This suggests that β3-AR activation may modulate the elastic properties of near-membrane components, such as the external basal membrane, sarcolemma, cytoskeletal network, and cytoplasm^50^. Further studies are still necessary to clarify the mechanisms by which CL316,243 lowers muscle stiffness and its potential role under pathological conditions associated with high muscle stiffness, including muscle dystrophies. Interestingly, β-adrenergic stimulation has been demonstrated to have also an acute effect on the myocardial stiffness of rabbits^51^. In particular, it has been found that exposure to isoprenaline, a non-selective β-AR agonist, induced a concentration-dependent reduction of myocardial stiffness in papillary muscles isolated from the rabbit’s ventricle. In this case, titin phosphorylation was hypothesized as the molecular mechanism responsible for the observed change in stiffness.

Together, these results suggest that targeting β3-AR may be an effective therapeutic strategy for enhancing muscle growth and strength in a variety of disorders associated with muscle loss and degeneration.

## Methods

### Animals and treatment with CL316,243

C57Bl/6 J wild type male mice aged 3 months were obtained from a breeding colony kept at the University of Naples and University of Catania. Mice were maintained at a controlled temperature (21 °C ± 1 °C) and humidity (50%) on a 12 h light/dark cycle (light from 06:00 to 18:00), with ad libitum food and water. All animal experimentation was conducted in accordance with the guidelines laid down by the European Community Council (2010/63/EU). The experimental protocols have been approved by the University Institutional Animal Care and Use Committee from the University of Naples (#0016945, 02/16/2012). Experiments were performed in parallel using 2 groups of mice treated with vehicle (n = 9) or CL316,243 (n = 10) at a concentration of 1 mg/kg in saline by subcutaneous injections for 15 days. Two hours after the last injection, mice underwent behavioral assessment of muscular strength by weights test^52^ and grip strength test^53^. Mice were then sacrificed by cervical dislocation and muscles were removed and frozen for western blot or processed for CSA evaluation.

### Behavioral assessment of muscle strength

The weights test was performed as previously described^52^. We used a series of chain links of different weight (from 14 to 74 gr) attached to a ball of fine wire mesh. Each mouse was held by the tail and was allowed to grasp a series of increasing weight steel chain links placed on the laboratory bench. Based on the number of chain links that the mouse was able to grasp and hold for at least 3 seconds, a specific score was assigned. If the mouse dropped the weight in less than 3 sec, the trial was repeated for 3 times and the maximum time/weight achieved was considered for the final scoring. After a rest period of about 20 sec, the next heaviest weight was tested until the mouse failed for 3 consecutive trials. A final total score was calculated as the product of the number of links in the heaviest chain held for the full 3 sec, multiplied by the time (sec) it was held. If the heaviest weight was dropped before 3 sec an appropriate intermediate value was calculated. For example, a mouse holding a 5-link weight for 3 seconds, but unable to lift a 6-link weight, was assigned a score of (5 × 3) = 15. If it held the 6-link weight for 1 second, the score was (5 × 3) + (1) = 16.

Grip strength test was performed as previously described^53^ by using an apparatus equipped with a mouse horizontal forelimb bar (Bioseb, Model GT3). Mice were held by the tail and were allowed to grasp the horizontal bar with the forelimb paws. The mice were then gently pulled backward until they released the grid. The peak force applied by the forelimbs of the mouse was recorded in Newton (N). Each mouse received 3 test trials (with a rest of 2 minutes) for two consecutive sessions (1 hour apart).

### Preparation and Culture of Muscle Fibers

Untreated adult mice (2–3 months old) were sacrificed by cervical dislocation and FDB muscles were quickly dissected and placed in a small petri dish filled with Tyrode Solution (in mM: 140 NaCl, 2 KCl, 2 CaCl_2_, 10 HEPES, and 5 glucose). FDB muscles were exposed to enzymatic digestion by using 0.2–0.3% Collagenase type I in Tyrode solution for 1 hour at 4 °C and then incubated in 5% CO_2_ for 1 hour at 37 °C. After three washes in Tyrode solution containing 10% FBS to block the collagenase effect and stabilize the fibers, FDB muscles were gently triturated to dissociate individual muscle fibers. The fibers were finally plated on laminin-coated culture dishes in serum-containing Tyrode solution and incubated in 5% CO_2_ at 37 °C until use.

### Immunofluorescence

For double immunofluorescence microscopy, the skeletal fibers in culture were fixed in 4% formaldehyde in PBS and permeabilized with PBS-Triton-X100 for 10 min at room temperature. Samples were then incubated for 4 hours with primary antibody against sarcomeric α-actinin (Sigma A-7811) in order to visualize Z-band on myofilaments. After washing in PBS, fibers were incubated for 1 hour in PBS 1% BSA with TRITC-conjugated goat anti-mouse (AlexaFluor 546). We used FITC-conjugated phalloidin (Sigma-Aldrich; St. Louis) to stain filamentous actin (F-actin) and 4′-6-diamidino-2-phenylindol (DAPI) for nuclei labeling. The specimens were visualized, with an Olympus IX-70 epifluorescence microscope equipped with a Hamamatzu-Orca ER II camera. Image ProPlus was used for the image acquisition.

### Atomic force microscopy

A commercial atomic force microscope (Keysight Technologies AFM model 5500) mounted on an inverted optical microscope (Olympus IX70) was used to assess the transversal stiffness of single dissociated skeletal muscle fibers^17^,^54^. Silicon cantilevers with a nominal spring constant k = 0.03 N/m and conical tips (CSC21, MikroMasch, Germany) were used. The spring constant of each cantilever was determined by a thermal noise based method, which ensures a level of accuracy of 10%–15%^55^. To probe the mechanical stiffness onto and below the sarcolemma, we performed force versus distance measurements and evaluated the contact region of the obtained curves. The transversal stiffness was measured as the Young’s modulus calculated by considering an approximate purely elastic response of the indented fiber, as proposed by Oliver and collaborators^56^. The stiffness was calculated at different penetration depths by applying controlled forces in the 0.5–2 nN range. Each measurement consisted of 256 force versus distance curves taken on the same 3 × 3 μm^2^ region. At least three measurements were performed on a single fiber for each time point (3 and 12 hours) and at least three different fibers were probed for each treated or control condition.

### Histology

Vehicle and CL316,243-treated mice were sacrificed by cervical dislocation and the limb muscles, TA and soleus, were harvested. Muscles were cross-cut, fixed in 10% buffered formalin, embedded in paraffin, then cut into 6 μm-thick serial sections and mounted on polylysine coated slides. Sections were stained with Hematoxylin and Eosin staining kit (Bio-optica, Milan, Italy), according to manufacturer protocol. Microscopic observation was performed by Leica DM2000LED (Leica Microsystems, Wetzlar, Germany) light microscope equipped with Leica ICC50HD digital camera for photodocumentation. Digital images acquired were then analyzed with SigmaScan Pro 5.0 software (SYSTAT, San Jose, CA, USA) to measure CSA. Measurements were performed by three independent observers and expressed as mean surface area (μm^2^). A total of 300–350 muscle fibers were analyzed for each muscle.

### Western blot analysis

TA and soleus of both vehicle and CL316,243-treated mice (n = 5 for each condition) were homogenized in lysis buffer (1:2, w/v) solution containing 0.5 M β-glycerophosphate, 20 mM MgCl2, 10 mM ethylene glycol tetraacetic acid, and supplemented with 100 mM dithiothreitol and protease/phosphatase inhibitors (100 mM dimethylsulphonyl fluoride, 2 mg/ml apronitin, 2 mM leupeptin, and 10 mM Na3VO4). Protein concentration was determined by the Bio-Rad protein assay (Bio-Rad, Milan, Italy). Samples containing 100 μg of proteins were denatured, separated on a 10% (for p70S6K and rpS6) or 8% (for nNOS) SDS-polyacrylamide gel, and electro-transferred onto a nitrocellulose membrane using a Bio-Rad Trans-Blot (Bio-Rad, Italy). Western blotting detection reagents were obtained from Amersham Biosciences (UK); the nitrocellulose membrane was from Hybond ECL (GE Healthcare, UK). Proteins were visualized by reversible staining with Ponceau S solution and destained in PBS^57^. Membranes were blocked at room temperature in milk buffer (1Χ PBS, 5–10% v/v non-fat dry milk, 0.2% v/v Tween-20) and then incubated at 4 °C overnight with the following primary antibodies: anti-phospho-p70^S6K^ (1:1000; Cell Signaling Technology, Massachusetts, USA), anti-phospho-rpS6 (1:1000; Cell Signaling Technology, Massachusetts, USA), anti-nNOS (1:300; Santa Cruz, California, USA); anti-α-tubulin antibody (1:1000; Cell Signaling Technology, Massachusetts, USA) or anti-GAPDH (1:8000; Sigma Aldrich, Milan, Italy). The membranes were then incubated for 90 min at room temperature with 1:5000 horseradish peroxidase-conjugated secondary anti-rabbit or anti-mouse antibodies. The resulting complexes were visualized using chemiluminescence Western blotting detection reagents. The western blot images were scanned using GS-800 imaging densitometer (Bio-Rad, Italy) and analyzed using Quantity One software (Biorad, Italy). The background-subtracted density of the bands in all blots was measured and normalized using α-tubulin or GAPDH.

### Statistical analysis

Data are presented as mean ± standard error of the mean (SEM). Statistical analyses were performed by using Systat software (Chicago, IL, USA). To compare the experimental conditions, we used unpaired Student’s t-test. A single TA and soleus muscle were examined for each treated animal. Two-way ANOVA was used for the AFM data. The level of significance was set at p < 0.05.

## Additional Information

**How to cite this article**: Puzzo, D. *et al*. CL316,243, a β3-adrenergic receptor agonist, induces muscle hypertrophy and increased strength. *Sci. Rep.* **6**, 37504; doi: 10.1038/srep37504 (2016).

**Publisher's note:** Springer Nature remains neutral with regard to jurisdictional claims in published maps and institutional affiliations.
